# Probable post-traumatic stress disorder and harmful alcohol use among male members of the British Police Forces and the British Armed Forces: a comparative study

**DOI:** 10.1080/20008198.2021.1891734

**Published:** 2021-03-25

**Authors:** Patricia Irizar, Sharon A.M. Stevelink, David Pernet, Suzanne H. Gage, Neil Greenberg, Simon Wessely, Laura Goodwin, Nicola T. Fear

**Affiliations:** a Department of Psychology, Institute of Population Health, University of Liverpool, UK; b King’s Centre for Military Health Research, Institute of Psychiatry, Psychology & Neuroscience, King’s College London, UK; c Department of Psychological Medicine, Institute of Psychiatry, Psychology & Neuroscience, King’s College London, UK; d Academic Department of Military Mental Health, Institute of Psychiatry, Psychology & Neuroscience, King’s College London, UK

**Keywords:** Harmful alcohol use, entropy balancing, police, mental health, military, post-traumatic stress disorder, uso nocivo de alcohol, balance de entropía, policía, salud mental, ejército, trastorno de estrés postraumático, 有害酒精使用, 熵平衡警察, 心理健康, 军事, 创伤后应激障碍。

## Abstract

**Background**: British Armed Forces’ and Police Forces’ personnel are trained to operate in potentially traumatic conditions. Consequently, they may experience post-traumatic stress disorder (PTSD), which is often comorbid with harmful alcohol use.

**Objective**: We aimed to assess the proportions, and associations, of probable PTSD and harmful alcohol use among a covariate-balanced sample of male military personnel and police employees.

**Methods**: Proportions of probable PTSD, harmful alcohol use, and daily binge drinking, were explored using data from the police Airwave Health Monitoring Study (2007–2015) (*N* = 23,826) and the military Health and Wellbeing Cohort Study (phase 2: 2007–2009, phase 3: 2014–2016) (*N* = 7,399). Entropy balancing weights were applied to the larger police sample to make them comparable to the military sample on a range of pre-specified variables (i.e. year of data collection, age and education attainment). Multinomial and logistic regression analyses determined sample differences in outcome variables, and associated factors (stratified by sample).

**Results**: Proportions of probable PTSD were similar in military personnel and police employees (3.67% vs 3.95%), although the large sample size made these borderline significant (Adjusted Odds Ratio (AOR): 0.84; 95% Confidence Intervals (CI): 0.72 to 0.99). Clear differences were found in harmful alcohol use among military personnel, compared to police employees (9.59% vs 2.87%; AOR: 2.79; 95% CI: 2.42 to 3.21). Current smoking, which was more prevalent in military personnel, was associated with harmful drinking and binge drinking in both samples but was associated with PTSD in military personnel only. **Conclusions**: It is generally assumed that both groups have high rates of PTSD from traumatic exposures, however, low proportions of PTSD were observed in both samples, possibly reflecting protective effects of unit cohesion or resilience. The higher level of harmful drinking in military personnel may relate to more prominent drinking cultures or unique operational experiences.

## HIGHLIGHTS

- Probable PTSD and harmful drinking were compared in military personnel and police employees.
- Proportions of probable PTSD were comparable in military personnel and police employees.
- Military personnel were three times more likely to drink harmfully than police employees.

## Abstract (es)

**Antecedentes**: El personal de las fuerzas armadas británicas y de la policía británica está entrenado para operar en condiciones potencialmente traumáticas. Consecuentemente, pueden experimentar trastorno de estrés postraumático (TEPT), el cual es frecuentemente comórbido con uso nocivo de alcohol.

**Objetivo**: Buscamos evaluar las proporciones, y asociaciones, del probable TEPT y del uso nocivo de alcohol en una muestra balanceada por covariables de personal masculino, militares y empleados de policía.

**Métodos**: Las proporciones de probable TEPT, uso nocivo de alcohol y atracones diarios de alcohol, fueron exploradas utilizando datos del estudio Airwave Health Monitoring Study de la policía (2007-2015) (*N*=23,826) y del estudio militar Health and Wellbeing Cohort Study (fase 2: 2007-2009, fase 3: 2014-2016) (*N*=7,399). Se aplicaron pesos de balance de entropía a la muestra más grande, de policía, para hacerla comparable a la muestra militar en un rango de variables pre-especificadas (ej. año de recolección de datos, edad y logros educacionales). Los análisis multinomiales y de regresión logística determinaron diferencias muestrales en las variables de resultado, y en los factores asociados (estratificados por muestra).

**Resultados**: Las proporciones de TEPT probable fueron similares en el personal militar y los empleados de policía (3,67% vs 3,95%), aunque el gran tamaño muestral hizo fuera significativo al límite (Razón de probabilidades ajustada (AOR): 0.84; Intervalo de Confianza (IC) de 95%: 0.72 a 0.99). Se encontraron claras diferencias en el uso nocivo de alcohol entre el personal militar, comparado a los empleados de policía (9,59% vs 2.87%; AOR: 2.79; IC 95%: 2.42 a 3.21). El consumo actual de tabaco, que fue más prevalente en el personal militar, se asoció a consumo nocivo de alcohol y a atracones de alcohol en ambas muestras, pero se asoció a TEPT sólo en el personal militar.

**Conclusiones**: Generalmente se asume que ambos grupos tienen altas tasas de TEPT desde la exposición traumática, sin embargo, se observó una baja proporción de TEPT en ambas muestras, lo que probablemente refleja el efecto protector de la cohesión de unidad o la resiliencia. El mayor nivel de consumo nocivo de alcohol en el personal militar puede estar relacionado una cultura de consumo de alcohol más prominente o a experiencias operacionales únicas.

## Abstract (zh)

**背景**:英国武装部队和警察部队的人员受训在潜在创伤情况下作战。因此, 他们可能会遭受创伤后应激障碍 (PTSD), 常与有害酒精使用共病。

**目的**:我们旨在评估一个男性军事人员和警务人员的协变量平衡样本中可能的PTSD和有害酒精使用的比例和关联。

**方法**:使用来自警方的《无线电波健康监测研究》 (2007年至2015年) (*N* = 23,826) 和《军事健康与福利研究》 (阶段2:2007-2009, 第3阶段:2014-2016) (*N* = 7,399) 的数据, 探索了可能的PTSD, 有害酒精使用和每日酗酒的比例。在较大的警察样本应用熵平衡权重, 使其在一系列预先指定变量 (即数据收集的年份, 年龄和受教育程度) 上与军事样本具有可比性。多项式和逻辑回归分析确定了结果变量中的样本差异以及相关因素 (按样本分层) 。

**结果**:军事人员和警务人员可能的PTSD比例相似 (3.67比3.95％), 尽管大样本量使差异边缘显著 (调整后的优势比 (AOR):0.84: 95％的置信区间 (CI):0.72至0.99) 。在有害酒精使用方面, 发现了军事人员与警务人员相比存在显著差异 (9.59％比2.87％: AOR:2.79: 95％CI:2.42至3.21) 。目前吸烟在军事人员中更为普遍, 在两个样本中都与有害饮酒和酗酒相关, 但仅与军事人员中的PTSD相关。

**结论**通常认为两群体都会因创伤暴露具有较高PTSD率, 但是, 在这两个样本中都观察到了较低比例的PTSD, 可能反映了单位凝聚力或心理韧性的保护作用。军事人员的有害饮酒水平更高, 可能与更突出的饮酒文化或独特的作战经验有关。

## 1. Introduction

The military and police respond rapidly during national, and international, disasters and conflicts, and often operate under high pressure, potentially being exposed to traumatic situations. Nevertheless, there may be some differences between these occupations in the types of exposure or type of traumatic experience. For example, military personnel may face intense stressors during set periods of time (e.g. deployment to a conflict situation), whereas for police employees the exposures may occur more regularly and in some cases be part of their daily routines. In terms of traumatic experiences, military personnel are more likely to have killed others, whereas police employees have to respond to and investigate civilian deaths and severe abuse of vulnerable others such as children (Hartley, Sarkisian, Violanti, Andrew, & Burchfiel, 2013; Osório et al., 2018; Stevelink et al., 2020). In addition, both groups may experience other causes of occupational stressors, such as competing demands with family life, demand-control imbalances or poor organizational support (Harvey et al., 2017, 2018). It is possible that varied nature of occupational and trauma stressors experienced by military personnel and police employees may have a differential impact on their mental health and patterns of alcohol consumption.

Prevalence estimates of adverse mental health outcomes among members of the UK Armed Forces are well documented as a result of a representative, longitudinal study set up to explore the impact of deployment to Iraq, and subsequently Afghanistan, on the health and wellbeing of military personnel (Fear et al., 2010; Hotopf et al., 2006; Stevelink et al., 2018). The most recent estimates from this cohort study indicate a prevalence of 6% for probable PTSD and 10% for alcohol misuse among military personnel (Stevelink et al., 2018). Most relevant mental health research concerning the UK police forces, has often been conducted in the aftermath of an emergency, and/or included only a select few police forces (Lawson, Rodwell, & Noblet, 2012; Maia et al., 2007; van der Velden et al., 2013). Though recently, a large UK survey suggested that of those police employees exposed to trauma, about one in five would develop symptoms of PTSD (Brewin, Miller, Soffia, Peart, & Burchell, 2020). Additionally, a recent meta-analysis identified a pooled global prevalence of 5% for harmful alcohol use among police employees and 14% for PTSD (Syed et al., 2020). However, an international review of hazardous and harmful drinking in trauma-exposed occupations, identified no UK studies of alcohol use in police officers (Irizar, Puddephatt, Fallon, Gage, & Goodwin, Under Review).

In this paper, we explore the proportions, and pre-specified associated factors, of probable PTSD and harmful alcohol use among covariate-balanced samples of male members of the British Armed Forces and the British Police Forces. In addition, we explore whether there is a difference in the comorbidity of probable PTSD and harmful alcohol consumption between the two samples. This study is pre-registered on Open Science Framework (DOI 10.17605/OSF.IO/7PTWX), where the research questions and data analyses plan are outlined in more detail.

## 2. Materials and methods

### 2.1. Study samples and data collection

#### 2.1.1. Airwave health monitoring study

Cross-sectional data on police employees was obtained from the Airwave Health Monitoring Study, which was established to determine possible health risks associated with the use of Terrestrial Trunked Radio (TETRA), a digital communication system used by emergency services since 2001 (Elliott et al., 2014). A total of 41,038 police employees completed measures relating to mental health and alcohol consumption, between June 2006 and March 2015. Out of the 54 existing police forces, 28 agreed to participate, with the response rates averaging 50% across participating forces. Participants completed an enrolment questionnaire including demographic, health and lifestyle items, and a health screen conducted by trained nurses. The Airwave Health Monitoring Study design and protocol have been described in detail in a previous publication (Elliott et al., 2014). We will refer to this sample as the ‘police sample’ throughout the rest of this paper.

**Figure 1.**
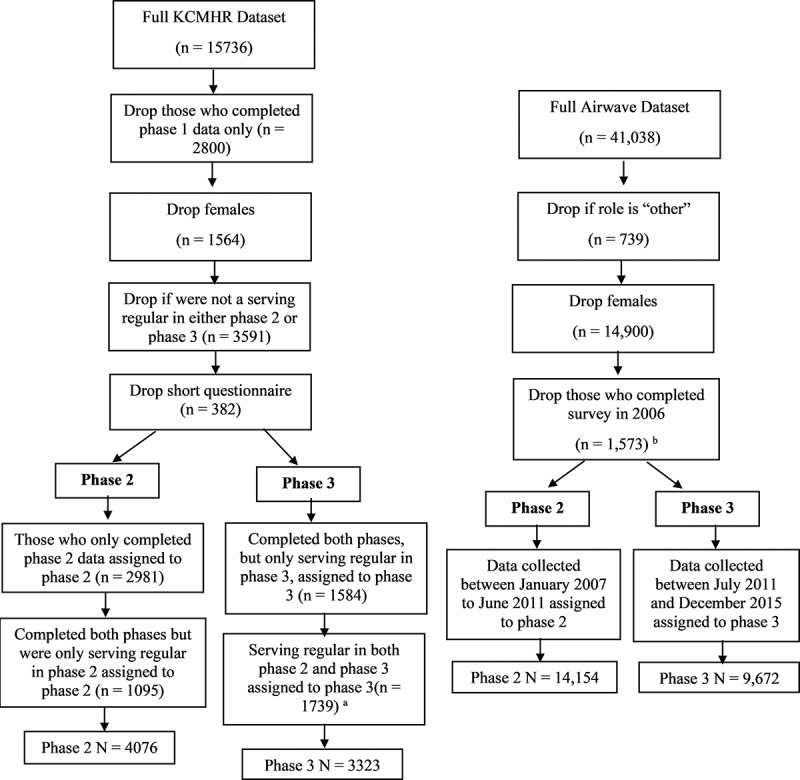
Flow diagram showing the allocation of participants to phase 2 and phase 3

^a^Participants with data for both phases were included in phase 3 to create a more equal distribution, which reflects the distribution of the Airwave data.^b^Participants who completed the Airwave Health Monitoring Study survey in 2006 were not asked the PTSD items, and so, were dropped. The phase 1 KCMHR data were also dropped as this was the same timeframe.

Participants were eligible for inclusion in the study if they identified themselves as an active member of the police force (e.g. inspector, police constable/sergeant or police staff). Those whose data were collected in 2006 were excluded as this version of the protocol did not include the measure of PTSD. This procedure is outlined in detail in Figure 1.

#### 2.1.2. Health and wellbeing cohort study

Data on UK military personnel was obtained from the health and wellbeing cohort study established by the King’s Centre for Military Health Research. Participants completed a self-administered questionnaire that was available in both hard copy and electronically (latest phase only), and included questions relating to demographics, service information, experiences during and returning from deployment, mental health, physical health and lifestyle. This cohort study was initially set up to investigate the impact of deployment to the conflict in Iraq on the health and wellbeing of military personnel (data collected between 2004–2006) (Hotopf et al., 2006) including a random sample of regular and reserve personnel of the UK Armed Forces, stratified by deployment status (phase 1; *n* = 10,272, response rate 59%). As the conflict in Iraq continued and personnel were also deployed to Afghanistan, all personnel included in phase 1 were asked to take part in phase 2 (data collected between 2007–2009). In addition, a random sample of personnel deployed to Afghanistan between April 2006 and April 2007 (termed the HERRICK sample), and a sample of newly trained personnel who joined the Armed Forces since April 2003 (termed the replenishment sample), were included (phase 2; *n* = 9990, response rate 56%) (Fear et al., 2010). An additional 300 responders were included who only filled in a short version of the phase 2 questionnaire after the main data collection for this phase had finished. Again, a replenishment sample of newly trained personnel who joined after June 2009 was included in phase 3 (data collected between 2014–2016), in addition to those who took part in phase 2 and agreed to future contact (phase 3; *n* = 8,093, response rate 57.8%) (Stevelink et al., 2018). The procedures for each phase are described in detail in previous publications (Fear et al., 2010; Hotopf et al., 2006; Stevelink et al., 2018). We will refer to this sample as the ‘military sample’ throughout the rest of the paper.

To ensure comparability with the police sample, only regular serving personnel were included from the military sample for which data was collected during phase 2 (2007–2009) and phase 3 (2014–2016) of the military health and wellbeing cohort study. Further, military personnel who filled in the short questionnaire at phase 2 were also dropped. Not all measures needed for the current comparative analysis were available. Females were also excluded for the purpose of this analysis (analysis will be repeated for females in a separate study); it is important to study males and females separately, as female members of both samples are relatively unique with regards to the type of role they can hold (particularly within the military) and have higher rates of mental health problems, than males (McManus, Bebbington, Jenkins, & Brugha, 2016). Further, the proportion of females is usually substantially higher in the police force (approx. 30%) (Allen & Audickas, 2020) than in the Armed Forces (approx. 10%) (Dempsey, 2019).

### 2.2. Measures

#### 2.2.1. Demographic, occupational and health variables

Comparable demographic, occupational, and health variables were obtained from both samples, including age, marital status, educational attainment, and smoking status. Income and police role (police staff, police constable/sergeant, inspector or above) were also obtained from the police sample. Type of Service at baseline (Naval Services, Army, Royal Air Force), rank (commissioned officer, non-commissioned officer or other) and deployment (yes/no), were obtained from the military sample.

#### 2.2.2. PTSD

In the police sample, probable PTSD was measured using the 10-item Trauma Screen.

Questionnaire (TSQ) (Brewin et al., 2002). Response options were on a five-point scale ranging from ‘not at all’ to ‘extremely’. Any responses other than ‘not at all’ were scored as 1 (score range 0–10). A score of 6 or more was defined as indicative of probable PTSD. The TSQ was only asked if participants responded positive to the following screening question: ‘Have you been bothered by a disturbing incident which has occurred over the past 6 months?’.

In the military sample, probable PTSD was measured using the 17-item National Centre for PTSD Checklist, civilian version (PCL-C) (Blanchard, Jones-Alexander, Buckley, & Forneris, 1996). Response options on the PCL-C are based on a five-point scale ranging from ‘not at all’ to ‘extremely’ (score range 17–85). A score of 50 or more was defined as indicative of probable PTSD. Research indicates that the PCL-C and TSQ, using the same defined cut-off scores as above, show similar prevalence estimates in the UK general population (McManus et al., 2016).

#### 2.2.3. Alcohol consumption

In the police sample, alcohol consumption was measured using a past week’s drinks diary, which asked participants to state the number of drinks they had consumed, for the following: white wine, red wine, fortified wine, spirits and beer (converted to units). In the military sample, the Alcohol Use Disorder Identification Test (AUDIT) (Saunders, Aasland, Babor, De la Fuente, & Grant, 1993) was used. To harmonize the measure of alcohol consumption with the police sample, two items were used (‘how often do you have a drink containing alcohol?’ and ‘how many units do you have on a typical day of drinking?’), to estimate total weekly units. AUDIT responses for the latter item are usually on a five-point scale, ranging from 1–2 drinks (scored as 0) to 10 or more drinks (scored as 4), but participants were given additional options, up to 30 or more, and provided responses in units. The frequency of consumption was multiplied with the midpoint for typical units, e.g. a participant who drinks two to three times a week and has 7 to 9 units on a typical day of drinking, would score 16 on total weekly units (2 × 8).

The UK’s Chief Medical Officer’s recommendation for weekly alcohol consumption was used to code both samples as ‘low-risk’ (≤14 units) and the National Institute for Health and Care Excellence (NICE) guidance for males was used to code anyone drinking above this as ‘hazardous’ (>14-50 units) and ‘harmful’ (>50 units) drinkers (Department of Health and Social Care, 2016; NICE, 2014). In the police sample, participants were asked if they currently drink alcohol; those who responded ‘no’ were categorized as ‘non-drinkers’. In the military sample, participants who responded with ‘never’ to the first item of the AUDIT, were categorized as ‘non-drinkers’.

Both datasets included a measure of binge drinking, i.e. ‘how often do you have six or more drinks on one occasion?’. Responses were given on a five-point scale; however, the comparability of this variable was reduced as the outcomes were worded slightly differently in each sample, except for ‘daily or almost daily’. Therefore, a binary variable, ‘binge drinks daily or almost daily’ vs ‘does not binge drink daily or almost daily’, was created to reflect more harmful drinking behaviours.

### 2.3. Statistical analysis

#### 2.3.1. Entropy balancing

Entropy balancing is a multivariate reweighting method, building on propensity score matching but allowing the full use of the larger sample (rather than selecting a matched sub-sample), used to achieve covariate balance on a range of pre-specified variables (year of data collection, age and educational attainment), to increase comparability (Hainmueller, 2012). Entropy balancing was used to create a weight value for all police employees, to be more comparable to the military dataset (which is smaller and more occupationally distinct), which is then used as a weight when estimating proportions in police employees.

A binary variable was created for year of data collection (January 2007-June 2011, reflecting phase 2 of the military sample, vs July 2011-December 2016, reflecting phase 3 of the military sample), using broad ranges rather than precise year to account for differences between the samples. Age was grouped into 10-year age bands, from <30 (starting at 18 and 20 years old for police employees and military personnel, respectively) up to ≥50 years old (up to 70 years old across both samples). Educational attainment was split into low (O levels/GCSEs or below) and high (A levels and equivalent or higher). There was some variation in the wording of the education question, as police employees were asked to report their qualifications at the time of the survey, and military personnel at the time of joining service. However, during research advisory groups, it was suggested that it is unlikely that police officers would gain education as part of their service, whereas military personnel may gain qualifications as part of their training. Entropy balancing was conducted in STATA using the ebalance command (Hainmueller & Xu, 2013).

#### 2.3.2. Estimating sample differences

Frequencies and percentages, with 95% confidence intervals (CI), were used to describe demographic, occupational and health variables for each sample. Descriptive statistics were also reported for the outcome variables, i.e. probable PTSD, alcohol consumption (non-drinkers, low risk, hazardous use and harmful use), binge drinking and comorbid probable PTSD and harmful alcohol consumption. The percentages were reported with entropy balance weights applied.

Stratifying by sample, logistic regressions (when PTSD and binge drinking were outcomes) and multinomial logistic regressions (when harmful alcohol use was the outcome, using low risk drinking as the reference group) were used to determine any associations between the demographic, occupational and health variables with PTSD and harmful alcohol use.

Logistic regressions were used to determine sample differences in probable PTSD and binge drinking. Multinomial logistic regressions determined sample differences in harmful alcohol use (low risk drinking as reference) and comorbidity of probable PTSD and harmful alcohol use (presence of neither as reference). Analyses were adjusted for variables hypothesized *a priori* to be associated with PTSD and harmful alcohol use: marital status and smoking status. Previous evidence shows that being married or in a relationship is protective against PTSD (Jakupcak et al., 2010) and harmful drinking (Prescott & Kendler, 2001), compared to those who are not in a relationship, whereas smoking has been found to be positively associated with PTSD (Fu et al., 2007) and harmful drinking (Room, 2004). We did not adjust for age and education as these variables were used to create the entropy balancing weight, though higher education is thought to be protective against PTSD and harmful drinking.

We conducted sensitivity analyses to explore whether the previously outlined associations differed if we restricted the police sample to inspectors, constables and sergeants, excluding police staff. Police staff may be less comparable to serving regular military personnel, as they are considered to have more desk-based duties compared to inspectors and police constables/sergeants.

Unadjusted and adjusted odds ratios or multinomial odds ratios, with 95% confidence intervals are reported. All statistical analyses were conducted in STATA SE 15.

### 2.4. Ethics

The Airwave Health Monitoring Study received ethical approval from the National Health Service multi-site research ethics committee (MREC/13/NW/0588). Written informed consent was obtained from all participants. Ethical approval was obtained for each of the phases of the Health and Wellbeing Cohort Study from both the UK Ministry of Defence Research Ethics Committee and the local Ethics Committee at King’s College London.

## 3. Results

### 3.1. Sample characteristics

The total sample size was 31,255, including 23,826 police employees and 7,399 military Personnel (Table 1). The entropy balancing resulted in balanced estimates of age and education whereby approximately 75% of both samples were under the age of 40 years and approximately 57% of both samples had a higher educational attainment. About 80% of police employees were constables and sergeants. Over 55% of military personnel were non-commissioned officers (NCOs) and had been on deployment. Almost 25% of military personnel reported smoking, compared to just 10% of police employees.

**Table 1.** Demographic (age, marital status, education, income), occupational (role, rank, deployment, service) and health (smoking status) characteristics from police (*N* = 23,826) and military personnel (*N* = 7,399)

		*Police*				*Military*			
*Characteristic*		*Total*	*N*	%	*95% CI*	*Total*	*N*	%	*95% CI*
Age (years)		23,651				7,399			
	< 29		2,469	35.71	34.64 to 36.79		2,896	39.18	38.06 to 40.31
	30 to 39		7,394	40.55	39.66 to 41.45		2,694	36.35	35.25 to 37.46
	40 to 49		10,193	21.09	20.56 to 21.63		1,428	19.26	18.37 to 20.18
	≥ 50		3,770	2.66	2.55 to 2.76		381	5.21	4.72 to 5.75
Marital status		23,240				7,298			
	Married/Cohabiting		19,747	79.06	78.19 to 79.92		5,764	79.00	78.04 to 79.93
	Divorced/Separated		1,619	5.18	4.83 to 5.55		395	5.32	4.82 to 5.86
	Single		1,874	15.76	14.94 to 16.61		1,139	15.68	14.86 to 16.54
Education		23,651				7,233			
	Low (GSCE/O level or below)		8,167	42.77	41.77 to 43.77		3,094	42.78	41.64 to 43.92
	High (Vocational/A levels or higher)		15,484	57.23	56.22 to 58.23		4,139	57.22	56.08 to 58.36
Smoking status		23,617				7,113			
	Non-smoker		21,681	89.98	89.36 to 90.56		5,481	75.48	74.47 to 76.47
	Current smoker		2,111	10.02	9.44 to 10.64		1,791	24.52	23.53 to 25.53
Income (police only)		23,651							
	Less than £25,999		2,096	15.49	14.62 to 16.41		-	-	-
	£26,000 – £37,999		9,655	48.47	47.49 to 49.44		-	-	-
	£38,000 – £59,999		10,832	34.09	33.25 to 34.95		-	-	-
	More than £60,000		1,068	1.94	1.79 to 2.12		-	-	-
Role (police only)		21,290							
	Police staff		3,627	15.28	14.53 to 16.07		-	-	-
	Police constable/sergeant		15,645	79.81	79.00 to 80.60		-	-	-
	Inspector or above		2,182	4.91	4.62 to 5.21		-	-	-
Rank (military only)						7,233			
	Other		-	-	-		1,717	21.47	20.54 to 22.43
	Non-commissioned officer		-	-	-		4,108	55.23	54.08 to 56.38
	Commissioned officer		-	-	-		1,574	23.30	22.34 to 24.28
Deployed (military only)						7,195			
	Not deployed		-	-	-		1,128	15.44	14.62 to 16.29
	Deployed		-	-	-		6,233	84.56	83.70 to 85.38
Service (military only)						7,399			
	Naval Services		-	-	-		1,197	16.18	15.47 to 17.17
	Army		-	-	-		4,751	64.21	62.70 to 64.92
	Royal Air Force		-	-	-		1,451	19.61	19.88 to 20.82

Percentages are weighted with entropy balancing (e.g. year of data collection, age and educational attainment).

Supplementary Table 1 compares the unweighted frequencies and percentages with the entropy balanced frequencies and percentages for the police sample. The weighted estimates for the outcomes of interest (i.e. PTSD, categories of alcohol consumption, binge drinking) were similar to the unweighted estimates. As expected, due to the covariate-balancing, the weighting increased the proportion of police employees under the age of 40 years and with lower educational attainment, similar to the military sample. The weighting lowered the proportion of inspectors and the proportion with a salary over £60,000 (See Supplementary Table 1).

### 3.2. Sample differences in probable PTSD and harmful alcohol use

For probable PTSD, 3.95% of police employees and 3.67% of military personnel met criteria. For harmful alcohol use, 2.87% of police employees and 9.59% of military personnel met criteria, with 1.50% of police employees and 3.04% of military personnel reporting daily or almost daily binge drinking. Military personnel were less likely to meet the criteria for probable PTSD compared to police employees (Adjusted Odds Ratio (AOR) 0.84, 95% CI 0.72 to 0.99), however this association was borderline significant only in the adjusted regression (Table 2). In contrast, military personnel were significantly more likely to report harmful alcohol use (AOR 2.79, 95% CI 2.42 to 3.21) compared to police employees and this is also reflected in their binge drinking behaviour (AOR 1.67, 95% CI 1.37 to 2.03). Military personnel were also more likely to report comorbid PTSD and harmful alcohol use (AOR 2.84, 95% CI 1.67 to 4.84), though this is likely driven by harmful alcohol use.

**Table 2.** Logistic and multinomial regression analyses showing the differences in PTSD and alcohol consumption characteristics stratified among police and military personnel. The police sample is the reference group ^a.^

*Outcome variable*		*Police* *N (%)*	*Military* *N (%)*	*OR (95% CI)*	*AOR (95% CI) ^b^*
PTSD					
	Non-case	22,721 (96.05)	7,009 (96.33)	1.00	1.00
	Case	944 (3.95)	266 (3.67)	0.93 (0.79 to 1.09)	0.84 (0.72 to 0.99)*
				*N = 30,749*	*N = 30,217*
Alcohol use (UK government guidelines)					
	Non-drinker	1,810 (8.52)	294 (3.93)	0.46 (0.40 to 0.54)***	0.46 (0.40 to 0.53)***
	Low risk (0 to 14 units)	11,656 (52.03)	3,754 (51.63)	1.00	1.00
	Hazardous (15 to 50 units)	9,429 (36.57)	2,524 (34.84)	0.96 (0.90 to 1.02)	0.90 (0.84 to 0.96)**
	Harmful (above 50 units)	767 (2.87)	683 (9.59)	3.37 (2.94 to 3.88)***	2.79 (2.42 to 3.21)***
				*N = 30,656*	*N = 30,143*
Binge drinking ^c^					
	No	23,169 (98.50)	7,075 (96.96)	1.00	1.00
	Yes	493 (1.50)	216 (3.04)	2.06 (1.71 to 2.48)***	1.67 (1.37 to 2.03)***
				*N = 30,672*	*N = 30,160*
Comorbidity					
	PTSD non-case and non-case harmful alcohol use	21,924 (93.41)	6,327 (87.58)	1.00	1.00
	PTSD case only	888 (3.71)	199 (2.77)	0.79 (0.67 to 0.95)*	0.75 (0.63 to 0.89)**
	Harmful alcohol use only	709 (2.62)	614 (8.69)	3.52 (3.07 to 4.04)***	3.02 (2.62 to 3.48)***
	PTSD case and harmful alcohol use case	55 (0.25)	61 (0.87)	3.76 (2.20 to 6.43)***	2.84 (1.67 to 4.84)***
				*N = 30,590*	*N = 30,083*

***p < .001, **p < .01, *p < .05. Percentages are weighted with entropy balancing (e.g. year of data collection, age and educational attainment).

^a^Age and education were not adjusted for as these variables were used in the entropy balancing to match the samples.

^b^Adjusted for marital status and smoking status.

^c^Binge drinking defined as drinking 6 or more units daily or almost daily.

The sensitivity analyses whereby the military personnel were compared to inspectors and police constables/sergeants only, and police staff were dropped, did not reveal any differences in the associations found between PTSD and alcohol consumption characteristics between military personnel and police employees (Supplementary Tables S3 and S4).

### 3.3. Associations with probable PTSD and alcohol consumption

Among both samples, greater educational attainment was significantly associated with decreased odds of PTSD (police employees: Odds Ratio (OR) 0.80, 95% Confidence Interval (CI) 0.65 to 0.98; military personnel: OR 0.69, 95% CI 0.54 to 0.89) (Table 3). Police employees aged ≥50 years (compared to those aged 30 to 39 years) had decreased odds of PTSD (OR 0.70, 95% CI 0.55 to 0.89). Among military personnel, being divorced/separated was significantly associated with increased odds of PTSD (OR 3.05, 95% CI 2.08 to 4.44,) and smoking (OR 1.95, 95% CI 1.51 to 2.53). Deployment reduced the likelihood of PTSD (OR 0.72, 95% CI 0.53 to 0.99) as well as holding a higher rank (NCOs OR 0.58, 95% CI 0.44 to 0.75, Commissioned Officers OR 0.30, 95% CI 0.19 to 0.46). Those in the Army were more likely to meet the criteria for PTSD, compared to those in the Royal Air Force or Naval Service.

**Table 3.** Demographic, occupational and health associations with PTSD caseness (PTSD non-case is the reference group) stratified by police and military personnel. Row percentages are shown representing the number of participants with probable PTSD

		PTSD Case			
		Police		Military	
*Explanatory variable*		*N caseness (%)*	*OR (95% CI)*	*N caseness (%)*	*OR (95% CI)*
Age (years)					
	< 29	85 (3.64)	0.87 (0.66 to 1.15)	120 (4.27)	1.23 (0.93 to 1.63)
	30 to 39	281 (4.15)	1.00	92 (3.50)	1.00
	40 to 49	463 (4.20)	1.01 (0.86 to 1.20)	46 (3.19)	0.91 (0.63 to 1.31)
	≥ 50	115 (2.95)	0.70 (0.55 to 0.89)**	8 (2.14)	0.60 (0.29 to 1.25)
Marital status					
	Married/Cohabiting	771 (3.91)	1.00	185 (3.23)	1.00
	Divorced/Separated	77 (3.79)	0.97 (0.68 to 1.37)	43 (3.90)	3.05 (2.08 to 4.44)***
	Single	73 (4.42)	1.14 (0.83 to 1.56)	35 (9.23)	1.22 (0.87 to 1.71)
Education					
	Low (GSCE/O level or below)	324 (4.44)	1.00	134 (4.42)	1.00
	High (Vocational/A levels or higher)	617 (3.57)	0.80 (0.65 to 0.98)*	127 (3.11)	0.69 (0.54 to 0.89)***
Smoking status					
	Non-smoker	871 (3.97)	1.00	161 (2.96)	1.00
	Current smoker	73 (3.75)	0.94 (0.64 to 1.38)	99 (5.62)	1.95 (1.51 to 2.53)***
Income (police only)					
	Less than £25,999	76 (3.75)	1.00	-	-
	£26,000 – £37,999	406 (4.16)	1.11 (0.78 to 1.59)	-	-
	£38,000 – £59,999	428 (3.74)	1.00 (0.71 to 1.42)	-	-
	More than £60,000	31 (3.40)	0.90 (0.49 to 1.68)	-	-
Role (police only)					
	Police constable/sergeants	661 (4.08)	1.00	-	-
	Police staff	104 (3.90)	0.95 (0.67 to 1.36)	-	-
	Inspector or above	94 (4.31)	1.06 (0.78 to 1.44)	-	-
Rank (military only)					
	Other	-	-	98 (5.87)	1.00
	Non-commissioned officer	-	-	140 (3.48)	0.58 (0.44 to 0.75)***
	Commissioned officer	-	-	28 (1.82)	0.30 (0.19 to 0.46)***
Deployed (military only)					
	Not deployed	-	-	52 (4.74)	1.00
	Deployed	-	-	213 (3.48)	0.72 (0.53 to 0.99)*
Service (military only)					
	Naval Services	-	-	33 (2.77)	0.45 (0.43 to 0.92)*
	Army	-	-	199 (4.30)	1.00
	Royal Air Force	-	-	34 (2.38)	0.54 (0.37 to 0.78)**

***p < .001, **p < .01, *p < .05. Percentages are weighted with entropy balancing (e.g. year of data collection, age and educational attainment).

For both samples, higher educational attainment reduced the odds of harmful alcohol use, whereas smoking and being over 40 years of age (compared to those aged 30 to 39 years) increased the odds of harmful alcohol use (Table 4). Harmful alcohol use was about twice as likely among police employees in the top two income brackets. Military personnel who had deployed had 2.14 times the odds (95% CI 1.64 to 2.81) of harmful alcohol use, compared to those who had not deployed. Higher ranked military personnel were less likely to report harmful alcohol use compared to other ranks.

**Table 4.** Demographic, occupational and health associations with harmful alcohol use (low risk drinking is reference group) and daily or almost daily binge drink (not binge drinking daily or almost daily is the reference group) stratified by police and military personnel. Row percentages are shown representing the number of participants drinking alcohol at harmful levels or reporting daily or almost daily binge drinking

		Harmful alcohol use				Daily or almost daily binge drinking			
		Police		Military		Police		Military	
Explanatory variable		*N* caseness (%)	OR (95% CI)	*N* caseness (%)	OR (95% CI)	*N* caseness (%)	OR (95% CI)	*N* caseness (%)	OR (95% CI)
Age (years)									
	< 29	61 (2.36)	0.95 (0.80 to 1.15)	383 (13.84)	1.25 (0.96 to 1.63)	13 (0.46)	0.25 (0.14 to 0.46)***	101 (3.64)	1.54 (1.12 to 2.13)**
	30 to 39	205 (2.93)	1.00	194 (7.46)	1.00	125 (1.83)	1.00	62 (2.39)	1.00
	40 to 49	357 (3.48)	1.16 (1.03 to 1.32)*	93 (6.77)	1.46 (1.03 to 2.07)*	245 (2.42)	1.33 (1.05 to 1.69)*	42 (3.05)	1.28 (0.86 to 1.91)
	≥ 50	144 (3.96)	1.20 (1.02 to 1.42)*	13 (3.46)	2.61 (1.21 to 5.63)*	110 (2.94)	1.63 (1.22 to 2.16)**	11 (2.93)	1.23 (0.64 to 2.36)
Marital status									
	Married/Cohabiting	637 (2.79)	1.00	417 (7.44)	1.00	426 (1.67)	1.00	150 (2.70)	1.00
	Divorced/Separated	56 (3.66)	1.44 (0.97 to 2.14)	61 (16.31)	2.71 (1.99 to 3.70)***	40 (0.57)	1.46 (0.93 to 2.30)	22 (5.82)	2.24 (1.42 to 3.56)***
	Single	60 (3.26)	1.23 (0.85 to 1.77)	202 (18.35)	3.66 (3.00 to 4.45)***	19 (2.42)	0.34 (0.19 to 0.61)***	44 (3.99)	1.51 (1.07 to 2.12)**
Education									
	Low (GSCE/O level or below)	311 (3.21)	1.00	395 (13.08)	1.00	223 (2.00)	1.00	124 (4.10)	1.00
	High (Vocational/A levels or higher)	451 (2.62)	0.79 (0.64 to 0.99)*	286 (7.01)	0.48 (0.41 to 0.57)***	267 (1.13)	0.56 (0.44 to 0.72)***	92 (2.25)	0.54 (0.41 to 0.71)***
Smoking status									
	Non-smoker	631 (2.54)	1.00	382 (7.14)	1.00	404 (1.24)	1.00	119 (2.23)	1.00
	Current smoker	135 (5.83)	2.90 (2.17 to 3.87)***	295 (17.02)	3.27 (2.75 to 3.89)***	89 (3.79)	3.13 (2.26 to 4.34)***	95 (5.48)	2.54 (1.93 to 3.35)***
Income (police only)									
	Less than £25,999	47 (1.83)	1.00	-	-	22 (0.06)	1.00	-	-
	£26,000 – £37,999	280 (2.62)	1.48 (0.97 to 2.24)	-	-	176 (1.22)	2.04 (1.11 to 3.74)**	-	-
	£38,000 – £59,999	403 (3.69)	2.31 (1.53 to 3.47)***	-	-	273 (2.27)	3.85 (2.13 to 6.96)***	-	-
	More than £60,000	32 (3.06)	2.07 (1.17 to 3.67)**	-	-	19 (1.98)	3.34 (1.44 to 7.74)***	-	-
Role (police only)									
	Police constable/sergeants	539 (3.02)	1.00	-	-	341 (1.61)	1.00	-	-
	Police staff	59 (2.82)	0.90 (0.65 to 1.24)	-	-	47 (2.01)	0.82 (0.55 to 1.23)	-	-
	Inspector or above	108 (2.22)	0.82 (0.56 to 1.19)	-	-	67 (1.33)	1.25 (0.82 to 1.91)	-	-
Rank (military only)									
	Other	-	-	240 (14.66)	1.00	-	-	69 (4.22)	1.00
	Non-commissioned officer	-	-	381 (9.66)	0.57 (0.47 to 0.68)***	-	-	111 (2.82)	0.66 (0.48 to 0.89)**
	Commissioned officer	-	-	62 (4.03)	0.23 (0.17 to 0.32)***	-	-	36 (2.34)	0.54 (0.36 to 0.82)**
Deployed (military only)									
	Not deployed	-	-	65 (5.97)	1.00	-	-	21 (1.93)	1.00
	Deployed	-	-	615 (10.27)	2.14 (1.64 to 2.81)***	-	-	192 (3.21)	1.69 (1.07 to 2.66)*
Service (military only)									
	Naval Services	-	-	130 (11.23)	1.25 (1.00 to 1.55)*	-	-	43 (3.70)	1.21 (0.86 to 1.72)
	Army	-	-	459 (10.12)	1.00	-	-	139 (3.07)	1.00
	Royal Air Force	-	-	94 (6.59)	0.61 (0.48 to 0.77)***	-	-	34 (2.39)	0.77 (0.53 to 1.13)

***p < .001, **p < .01, *p < .05. Percentages are weighted with entropy balancing (e.g. year of data collection, age and educational attainment).

Interestingly, younger military personnel (<29) were more likely binge drink compared to those aged 30 to 39, but younger police employees were less likely to binge drink (Table 4). For both samples, binge drinking was less likely among those with higher educational attainment and more likely among current smokers. Police employees who reported to be single were less likely to binge drink (OR 0.34, 95% CI 0.19 to 0.61) compared to their married/cohabiting peers. In contrast, single, divorced and separated military personnel had an increased odds of binge drinking compared to their married/cohabiting counterparts. Military personnel who had deployed had a higher odds of binge drinking compared to those who had not deployed (OR 1.69, 95% CI 1.07 to 2.66).

## 4. Discussion

### 4.1. Key findings

This is the first study to directly compare the proportions, and associated factors, of probable PTSD and harmful alcohol use, among male members of the Armed Forces and Police Forces. We found several key findings. First, similar proportions of probable PTSD (approx. 4%) in both samples. Second, military personnel reported higher proportions of harmful alcohol use than police employees (approx. 10% vs 3%, respectively). Third, comorbid PTSD and harmful alcohol use were more common in military personnel compared to police employees, driven by the higher proportion of harmful alcohol use. In military personnel, holding a lower rank, being in the Army, divorced or separated or a current smoker all increased the likelihood of probable PTSD. Interestingly, the risk of binge drinking was higher in the youngest military personnel but higher in older police employees. Higher educational attainment was protective against probable PTSD, harmful alcohol use and daily binge drinking in both samples. Likewise, military personnel with lower ranks were more likely to drink harmfully. But contrarily, this was true for police employees with higher, not lower, salaries.

### 4.2. Proportions of probable PTSD, harmful alcohol use and binge drinking

Whilst there was a borderline significantly lower proportion of PTSD among military personnel than police employees, the proportions were similar (3.67% vs 3.95%). Both estimates are lower than observed in the UK general population (4.4%, using the PCL-C) (McManus et al., 2016) and lower than the most recent figures from the full cohort from which the military sample was derived (6.2%) (Stevelink et al., 2018). However, the current sample included only serving regular military personnel with the full military cohort showing higher proportions of probable PTSD in ex-serving and reserve personnel (Stevelink et al., 2018). Personnel with poor mental health are more likely to leave service and have poorer outcomes after leaving (Buckman et al., 2013; Iversen et al., 2005). We observed lower proportions of probable PTSD in police employees than other UK and international studies, as a recent study identified that 8% met criteria for PTSD (Brewin et al., 2020), and a systematic review estimated that 14% of police employees develop PTSD (Syed et al., 2020). However, these studies were based on occupational surveys, focussing on traumatic exposures, mental health and working conditions, which we have suggested causes a non-random bias from framing effects, increasing the reported prevalence of mental health disorders (Goodwin et al., 2013), whereas current data were obtained using a non-mental health focussed survey.

Our finding that the proportions of probable PTSD in both the Armed Forces and Police Forces were lower than those in the general population is of interest. This may be because both use stringent employment selection procedures and medical screening (Royal Navy, 2016; Violanti et al., 2017), which may lead to a more resilient workforce, otherwise known as the ‘healthy worker effect’ (Li & Sung, 1999). A recent review identified that the rates of PTSD among police officers were lower than for civilians who experienced similar traumatic events (Regehr et al., 2019), indicating that police employees may be more resilient, following trauma-exposure (Andrew et al., 2008). This could be because police employees and military personnel are trained to operate in traumatic situations, therefore trauma is an expected part of their role and experienced collectively, rather than individually as with civilians, with evidence showing that team and supervisory support is protective of mental health, in military personnel (Jones et al., 2012). Further, there may be selection bias as those who experience poor mental health are more likely to leave service early (Buckman et al., 2013), or reporting bias as these occupational groups may underreport their symptoms due to stigma and barriers to care (Haugen, McCrillis, Smid, & Nijdam, 2017; Sharp et al., 2015).

We found that the proportions of harmful alcohol use, and daily binge drinking, were three times greater in military personnel compared to police employees, with similarly increased proportions of comorbid PTSD and harmful alcohol use. Approximately 10% of military personnel met criteria for harmful alcohol use whereas only 3% of police employees did so, which is similar to males in the UK general population (Digital, 2018). The higher proportions of harmful drinking in military personnel may relate to coping motivations for drinking, hence the higher proportions of comorbid PTSD and harmful alcohol use. Recent findings showed that military personnel who drink to cope are more likely to drink harmfully and binge drink (Irizar, Leightley et al., 2020). Alcohol use has historically been part of military culture, often used to create social bonds or to destress following deployment (Ames, Cunradi, Moore, & Stern, 2007; Jones & Fear, 2011). In addition, ‘dry periods’ during operational deployment may make it more difficult to manage alcohol use upon return (Goodwin et al., 2017; Jacobson et al., 2008; Stevelink et al., 2018), though deployment can be protective against dependence. Military organizations are referred to as ‘greedy institutions’, with extensive demands for commitment and dedication, leading to the development of a strong military identity and group cohesion, with traditions and rituals encouraging heavy drinking to increase social bonding within the unit (Hatch et al., 2013). Alcohol has also been seen as an integral part of police culture, through social ponding rituals or to cope with the stress of the job, as drinking with colleagues provides an opportunity for an informal debriefing of shared experiences (Abdollahi, 2002; Richmond, Kehoe, Hailstone, Wodak, & Uebel‐Yan, 1999; Violanti, 2004). However, it is possible that the drinking culture has shifted in police employees, more so than military personnel, due to the ‘greedy’ nature of the military institution.

### 4.3. Factors associated with probable PTSD, harmful alcohol use and binge drinking

There were sample differences in the factors associated with harmful drinking and binge drinking. Police employees with higher salaries (indicating higher rank) were more likely to drink harmfully, and contrarily, military personnel holding a lower rank were more likely to drink harmfully. Moreover, the youngest police employees were less likely to binge drink compared to the older age groups, but the youngest military personnel were more likely to binge drink (consistent with the UK general population (Kuntsche, Kuntsche, Thrul, & Gmel, 2017)). The pattern of harmful drinking in police employees is similar to that of the general population, which shows that alcohol use is decreasing in younger people but increasing in older people (Bardsley et al., 2018; Oldham et al., 2020). Younger military personnel are more likely than those over 50 to report social pressure motivations for drinking (Irizar, Leightley et al., 2020) and may be more susceptible to military culture, which has historically facilitated risky drinking (Ames et al., 2007; Jones & Fear, 2011).

Smoking, which was more prevalent in military personnel than police employees (25% vs 10%), was only associated with probable PTSD in military personnel. Smoking was associated with harmful drinking and binge drinking in both samples, replicating findings from the full military cohort, though smoking rates have declined (Hooper et al., 2008; Thandi et al., 2015). In both samples, higher educational attainment was protective against all outcomes (PTSD, harmful drinking and binge drinking), in line with existing literature (A. C. Iversen et al., 2008; Jones et al., 2015; Tang, Deng, Glik, Dong, & Zhang, 2017). In military personnel, holding a higher rank and deployment were associated with a lower risk of PTSD, in congruence with previous findings from the first two phases of the cohort study, though deployment was associated with an increased risk of PTSD in the third phase of the cohort study (Stevelink et al., 2018), but this was more likely in reserves than regular serving personnel. The relationship between deployment and PTSD is complex and there are several interacting factors, such as whether personnel held a combat role and number of deployments (Jones et al., 2013; Sundin, Fear, Iversen, Rona, & Wessely, 2010).

### 4.4. Strengths and limitations

This study utilized two large samples with good response rates (above 50%). Using entropy balancing, we increased the comparability of the samples by balancing covariate distribution of variables known to be associated with the outcome variables across all participants, unlike methods such as propensity matching. Further, we were able to harmonize several variables which had variations, including the categorization of alcohol consumption and daily binge drinking. Nevertheless, a previous study has shown that higher quantities of alcohol are reported in a weekly drinks diary, as used in our police sample, compared to quantity-frequency measures (e.g. AUDIT) as used in our military sample (Heeb & Gmel, 2005). However, our study included additional response options for our military sample to capture higher quantities (up to 30 drinks or more), preventing an upper limit on weekly drinks. Another caveat is that police employees only completed the TSQ if they experienced a traumatic stressor in the past 6 months, possibly missing participants with chronic or delayed onset PTSD (Utzon-Frank et al., 2014), and reducing the comparability with the military sample, where the timeframe of the PCL-C was not limited. In our pre-registered protocol, we stated that we would harmonize the measures of probable PTSD by selecting 10 items of the PCL-C which were most comparable to the TSQ, but due to different scoring approaches we were unable to do this, and so the full PCL-C and TSQ were used for the military sample and police sample respectively, though both scales show similar prevalence estimates in the UK general population (McManus et al., 2016). However, there are currently no formal comparisons of the TSQ and PCL-C, other than comparing prevalence estimates, so the validity of this approach is unknown. Despite these incompatibilities, the samples were carefully matched, both manually (i.e. selecting male serving regulars only) and statistically (i.e. balancing covariates), to increase comparability.

### 4.5. Implications

The current emphasis on ensuring support is available for both the Armed Forces and Police Forces should continue, through ‘active monitoring’ for those who have been exposed to trauma and further help being accessible to those who need it (Greenberg, Megnin-Viggars, & Leach, 2019). Further, a brief screen for harmful alcohol use should be integrated with mental health support, as those who use alcohol to cope may experience further mental health decline (Strid, Andersson, & Öjehagen, 2018), which is particularly important for military personnel, given the higher rates of harmful drinking and comorbid PTSD and harmful drinking.

## 5. Conclusions

We identified comparable proportions of probable PTSD in a covariate-balanced sample of male UK military personnel and police employees and much higher proportion of harmful alcohol use in military personnel, compared with police employees. The latter findings highlight a need for evidence-based interventions, such as brief alcohol screens, which are effective in civilian occupational settings (Hermansson, Helander, Brandt, Huss, & Rönnberg, 2010), in military occupational settings, more so than other occupational groups. Low proportions of PTSD were observed in both samples, which could be indicative of protective effects of unit cohesion and resilient workforces. These factors should be explored further.

## Supplementary Material

Supplemental Material

## Data Availability

The police employee data underlying the results presented in the study are available to researchers who apply for access to the Airwave Health Monitoring Study via the following URL: https://police-health.org.uk/applying-access-resource. The military personnel data is not publicly available.

## Data availability statement

The data described in this article are openly available in the Open Science Framework at https://doi.org/10.17605/OSF.IO/TPA6U.

## Open Scholarship

This article has earned the Center for Open Science badge for Preregistered. The materials are openly accessible at https://doi.org/10.17605/OSF.IO/TPA6U.
