# Supplementary material for: Probable post-traumatic stress disorder and harmful alcohol use among male members of the British Police Forces and the British Armed Forces: a comparative study
Source: Eur J Psychotraumatol. 2021 Mar 25;12(1):1891734. doi: 10.1080/20008198.2021.1891734 (PMC8079084; doi:10.1080/20008198.2021.1891734)
Supplement: Supplemental Material [file ZEPT_A_1891734_SM6942.docx]

**Supplementary Table 1.** Demographic, physical health, mental health and alcohol characteristics for police personnel before and after entropy balancing.

|  | |  | *Police (representative estimates)* | | *Police (entropy balanced)* | |  |
| --- | --- | --- | --- | --- | --- | --- | --- |
| *Characteristic* | | *N* | % | *95% CI* | % | *95% CI* |  |
| Age (years) | |  |  |  |  |  |  |
|  | < 29 | 2,469 | 10.36 | 9.98 to 10.76 | 35.71 | 34.63 to 36.79 |  |
|  | 30 to 39 | 7,394 | 31.03 | 30.45 to 31.62 | 40.55 | 39.66 to 41.45 |  |
|  | 40 to 49 | 10,193 | 42.78 | 42.15 to 43.41 | 21.09 | 20.56 to 21.63 |  |
|  | ≥ 50 | 3,770 | 15.82 | 15.37 to 16.29 | 2.66 | 2.55 to 2.76 |  |
| Marital status | |  |  |  |  |  |  |
|  | Married/Cohabiting | 19,747 | 84.97 | 84.50 to 85.42 | 79.06 | 78.19 to 79.92 |  |
|  | Divorced/Separated | 1,619 | 6.97 | 6.64 to 7.30 | 15.76 | 14.94 to 16.61 |  |
|  | Single | 1,874 | 8.06 | 7.72 to 8.42 | 5.18 | 4.83 to 5.55 |  |
| Education | |  |  |  |  |  |  |
|  | Low (GSCE/O level or below) | 8,167 | 34.53 | 33.93 to 35.14 | 42.77 | 41.79 to 43.76 |  |
|  | High (Vocational/A levels or higher) | 15,484 | 65.47 | 64.86 to 66.07 | 57.23 | 56.24 to 58.21 |  |
| Smoking status | |  |  |  |  |  |  |
|  | Non-smoker | 21,681 | 91.12 | 90.76 to 91.48 | 89.98 | 89.36 to 90.56 |  |
|  | Current smoker | 2,111 | 8.87 | 8.52 to 9.24 | 10.02 | 9.44 to 10.64 |  |
| Income (police only) | |  |  |  |  |  |  |
|  | Less than £25999 | 2,096 | 8.86 | 8.51 to 9.23 | 14.17 | 13.42 to 14.95 |  |
|  | £26000 - £37999 | 9,655 | 40.82 | 40.20 to 41.45 | 50.51 | 49.55 to 51.47 |  |
|  | £38000 – £59999 | 10,832 | 45.80 | 45.17 to 46.43 | 33.37 | 32.54 to 34.20 |  |
|  | £More than £60000 | 1,068 | 4.52 | 4.26 to 4.79 | 1.95 | 1.80 to 2.12 |  |
| Role (police only) | |  |  |  |  |  |  |
|  | Police staff | 3,627 | 16.91 | 16.41 to 17.41 | 15.28 | 14.53 to 16.07 |  |
|  | Police constable/sergeants | 15,645 | 72.92 | 72.32 to 73.51 | 79.81 | 79.00 to 80.60 |  |
|  | Inspector or above | 2,182 | 10.17 | 9.77 to 10.58 | 4.91 | 4.62 to 5.21 |  |
| PTSD | |  |  |  |  |  |  |
|  | Non-case | 22,721 | 96.01 | 95.75 to 96.25 | 96.05 | 95.66 to 96.42 |  |
|  | Case | 944 | 3.99 | 3.75 to 4.25 | 3.95 | 3.58 to 4.34 |  |
| Alcohol use (UK government guidelines) | |  |  |  |  |  |  |
|  | Low risk (0 to 14 units) | 11,656 | 49.26 | 48.62 to 49.90 | 52.03 | 51.07 to 52.99 |  |
|  | Non-drinker | 1,810 | 7.65 | 7.32 to 8.00 | 8.52 | 7.98 to 9.10 |  |
|  | Hazardous (15 to 50 units) | 9,429 | 39.85 | 39.23 to 40.47 | 36.57 | 35.67 to 37.48 |  |
|  | Harmful (above 50 units) | 767 | 3.24 | 3.02 to 3.47 | 2.87 | 2.59 to 3.19 |  |
| Binge drinking | |  |  |  |  |  |  |
|  | No | 23,169 | 97.92 | 97.73 to 98.09 | 98.50 | 98.31 to 98.68 |  |
|  | Yes | 493 | 2.08 | 1.91 to 2.27 | 1.50 | 1.32 to 1.69 |  |
| Comorbidity | |  |  |  |  |  |  |
|  | PTSD non-case and non-case harmful alcohol use | 21,924 | 92.99 | 92.66 to 93.31 | 93.41 | 92.93 to 93.86 |  |
|  | PTSD case only | 888 | 3.77 | 3.53 to 4.02 | 3.71 | 3.37 to 4.10 |  |
|  | Harmful alcohol use only | 709 | 3.01 | 2.80 to 3.23 | 2.63 | 2.36 to 2.92 |  |
|  | PTSD case and harmful alcohol use case | 55 | 0.23 | 0.18 to 0.30 | 0.25 | 0.15 to 0.39 |  |

**Supplementary Table 2.** Logistic and multinomial logistic regression analyses showing the differences in PTSD and alcohol consumption characteristics among police and military personnel, without entropy balancing weights applied. The police sample is the reference group.

| *Outcome variable* | | *Total N in adjusted model* | *Police*  *N (%)* | *Military*  *N (%)* | *OR (95% CI)* | *AOR (95% CI)^a^* |
| --- | --- | --- | --- | --- | --- | --- |
| PTSD | | 30,360 |  |  |  |  |
|  | Non-case |  | 22,721 (96.01) | 7,009 (96.34) | 1.00 | 1.00 |
|  | Case |  | 944 (3.99) | 266 (3.66) | 0.91 (0.80 to 1.05) | 0.87 (0.76 to 1.01) |
|  |  |  |  |  | *N = 30,940* | *N = 30,360* |
| Alcohol use (UK government guidelines) | | 30,285 |  |  |  |  |
|  | Low risk (0 to 14 units) |  | 11,656 (49.26) | 3,754 (51.74) | 1.00 | 1.00 |
|  | Non-drinker |  | 1,810 (7.65) | 294 (4.05) | 0.50 (0.44 to 0.57)*** | 0.47 (0.41 to 0.54)*** |
|  | Hazardous (15 to 50 units) |  | 9,429 (39.85) | 2,524 (34.79) | 0.83 (0.78 to 0.88)*** | 077 (0.73 to 0.82)*** |
|  | Harmful (above 50 units) |  | 767 (3.24) | 683 (9.41) | 2.76 (2.48 to 3.08)*** | 2.15 (1.92 to 2.41)*** |
|  |  |  |  |  | *N = 30,917* | *N = 30,285* |
| Binge drinking ^b^ | | 30,304 |  |  |  |  |
|  | No |  | 23,169 (97.92) | 7,075 (97.03) | 1.00 | 1.00 |
|  | Yes |  | 493 (2.08) | 216 (2.97) | 2.06 (1.71 to 2.48)*** | 1.22 (1.03 to 1.45)* |
|  |  |  |  |  | *N = 30,935* | *N = 30,304* |
| Comorbidity | | 30,224 |  |  |  |  |
|  | PTSD non-case and non-case harmful alcohol use |  | 21,924 (92.99) | 6,327 (87.86) | 1.00 | 1.00 |
|  | PTSD case only |  | 888 (3.77) | 199 (2.76) | 0.78 (0.66 to 0.91)** | 0.76 (0.65 to 0.89)** |
|  | Harmful alcohol use only |  | 709 (3.01) | 614 (8.53) | 3.00 (2.68 to 3.36)*** | 2.43 (2.16 to 2.74)*** |
|  | PTSD case and harmful alcohol use case |  | 55 (0.23) | 61 (0.85) | 3.84 (2.67 to 5.54)*** | 3.01 (2.05 to 4.42)*** |
|  |  |  |  |  | *N = 30,777* | *N = 30,224* |

***p<.001, **p<.01, *p<.05

^a^ adjusted for marital status and smoking status.

^b^ Binge drinking defined as drinking 6 or more units daily or almost daily.

**Supplementary Table 3.** Sensitivity analysis showing the demographic, occupational and health associations with PTSD caseness, harmful alcohol use and daily binge drinking, in police employees, excluding police staff (N = 20,235).

|  | | *PTSD Case* | | *Harmful Alcohol Use* | | *Daily binge drinking* | |
| --- | --- | --- | --- | --- | --- | --- | --- |
| *Explanatory variable* | | *N (%)* | *OR (95% CI)* | *N (%)* | *OR (95% CI)* | *N (%)* | *OR (95% CI)* |
| Age (years) | |  |  |  |  |  |  |
|  | < 29 | 68 (3.41) | 0.80 (0.60 to 1.09) | 48 (2.35) | 0.73 (0.51 to 1.04) | 10 (0.45) | 0.25 (0.12 to 0.49)*** |
|  | 30 to 39 | 259 (4.21) | 1.00 | 180 (2.90) | 1.00 | 107 (1.79) | 1.00 |
|  | 40 to 49 | 433 (4.34) | 1.03 (0.87 to 1.22) | 329 (3.54) | 1.32 (1.08 to 1.62)** | 231 (2.53) | 1.43 (1.11 to 1.83)** |
|  | ≥ 50 | 80 (3.32) | **0.78 (0.59 to 1.03)** | 102 (4.47) | 1.66 (1.26 to 2.19)*** | 78 (3.27) | 1.86 (1.34 to 2.57)*** |
| Marital status | |  |  |  |  |  |  |
|  | Married/Cohabiting | 691 (3.86) | 1.00 | 548 (2.76) | 1.00 | 371 (1.67) | 1.00 |
|  | Divorced/Separated | 60 (4.63) | 1.21 (0.85 to 1.72) | 50 (3.94) | 0.94 (0.68 to 1.28) | 33 (2.28) | 1.38 (0.85 to 2.23) |
|  | Single | 67 (3.87) | 1.00 (0.70 to 1.44) | 48 (3.37) | 1.30 (0.99 to 1.69) | 14 (0.59) | 0.35 (0.18 to 0.66)** |
| Education | |  |  |  |  |  |  |
|  | Low (GSCE/O level or below) | 285 (4.29) | 1.00 | 271 (3.36) | 1.00 | 195 (2.10) | 1.00 |
|  | High (Vocational/A levels or higher) | 552 (3.69) | **0.85 (0.69 to 1.06)** | 383 (2.51) | 0.71 (0.56 to 0.90)** | 228 (1.07) | 0.50 (0.39 to 0.66)*** |
| Smoking status | |  |  |  |  |  |  |
|  | Non-smoker | 777 (3.99) | 1.00 | 544 (2.58) | 1.00 | 350 (1.26) | 1.00 |
|  | Current smoker | 63 (3.60) | 0.90 (0.60 to 1.35) | 114 (5.71) | 2.95 (2.15 to 4.05)*** | 76 (3.87) | 3.15 (2.19 to 4.52)*** |
| Income (police only) | |  |  |  |  |  |  |
|  | Less than £25999 | 36 (3.38) | 0.82 (0.52 to 1.29) | 14 (1.33) | 0.51 (0.26 to 1.00) | 4 (0.38) | 0.31 (0.10 to 0.93)* |
|  | £26000 - £37999 | 363 (4.11) | 1.00 | 237 (2.57) | 1.00 | 153 (1.20) | 1.00 |
|  | £38000 – £59999 | 409 (3.86) | 0.94 (0.76 to 1.15) | 376 (3.71) | 1.60 (1.25 to 2.03)*** | 251 (2.22) | 1.86 (1.40 to 2.46)*** |
|  | More than £60000 | 29 (3.47) | 0.84 (0.47 to 1.48) | 27 (2.75) | **1.29 (0.79 to 2.11)** | 15 (1.89) | **1.58 (0.76 to 3.31)** |
| Role (police only) | |  |  |  |  |  |  |
|  | Police constable/sergeants | 661 (4.07) | 1.00 | 539 (3.02) | 1.00 | 341 (1.59) | 1.00 |
|  | Inspector or above | 94 (4.32) | 1.06 (0.78 to 1.45) | 544 (2.24) | 0.82 (0.56 to 1.20) | 47 (2.01) | 1.27 (0.83 to 1.94) |

***p<.001, **p<.01, *p<.05. Percentages are weighted with entropy balancing (e.g. year of data collection, age and educational attainment).

**Supplementary Table 4.** Sensitivity regression analyses showing the differences in PTSD and alcohol consumption, stratified among police and military personnel (excluding police staff). The police sample is the reference group.

| *Outcome variable* | | *Police*  *N (%)* | *Military*  *N (%)* | *OR (95% CI)* | *AOR (95% CI) ^b^* |
| --- | --- | --- | --- | --- | --- |
| PTSD | |  |  |  |  |
|  | Non-case | 19,222 (96.05) | 7,009 (96.33) | 1.00 | 1.00 |
|  | Case | 840 (3.94) | 266 (3.67) | 0.93 (0.79 to 1.09) | 0.84 (0.71 to 0.99)* |
|  |  |  |  | *N = 27,149* | *N = 26,695* |
| Alcohol use (UK government guidelines) | |  |  |  |  |
|  | Low risk (0 to 14 units) | 9,822 (51.81) | 3,754 (51.63) | 1.00 | 1.00 |
|  | Non-drinker | 1,418 (8.01) | 294 (3.93) | 0.49 (0.42 to 0.57)*** | 0.49 (0.42 to 0.57)*** |
|  | Hazardous (15 to 50 units) | 8,166 (37.30) | 2,524 (34.84) | 0.94 (0.88 to 1.00) | 0.87 (0.81 to 0.93)*** |
|  | Harmful (above 50 units) | 659 (2.88) | 683 (9.59) | 3.35 (2.90 to 3.87)*** | 2.71 (2.34 to 3.14)*** |
|  |  |  |  | *N = 27,075* | *N = 26,639* |
| Binge drinking ^c^ | |  |  |  |  |
|  | No | 19,639 (98.49) | 7,075 (96.96) | 1.00 | 1.00 |
|  | Yes | 426 (1.51) | 216 (3.04) | 2.04 (1.69 to 2.47)*** | 1.65 (1.34 to 2.02)*** |
|  |  |  |  | *N = 27,091* | *N = 26,656* |
| Comorbidity | |  |  |  |  |
|  | PTSD non-case and non-case harmful alcohol use | 18,547 (93.41) | 6,327 (87.58) | 1.00 | 1.00 |
|  | PTSD case only | 791 (3.72) | 199 (2.77) | 0.79 (0.66 to 0.95)* | 0.75 (0.62 to 0.89)** |
|  | Harmful alcohol use only | 608 (2.63) | 614 (8.69) | 3.51 (3.04 to 4.06)*** | 2.95 (2.55 to 3.42)*** |
|  | PTSD case and harmful alcohol use case | 48 (0.24) | 61 (0.87) | 3.82 (2.14 to 6.80)*** | 2.91 (1.65 to 5.10)*** |
|  |  |  |  | *N = 27,011* | *N = 26,581* |

***p<.001, **p<.01, *p<.05. Percentages are weighted with entropy balancing (e.g. year of data collection, age and educational attainment).

^a^ Age and education were not adjusted for as these variables were used in the entropy balancing to match the samples.

^b^ Adjusted for marital status and smoking status.

^c^ Binge drinking defined as drinking 6 or more units daily or almost daily.
